# Metal ions shape α-synuclein

**DOI:** 10.1038/s41598-020-73207-9

**Published:** 2020-10-01

**Authors:** Rani Moons, Albert Konijnenberg, Carl Mensch, Roos Van Elzen, Christian Johannessen, Stuart Maudsley, Anne-Marie Lambeir, Frank Sobott

**Affiliations:** 1 Biomolecular and Analytical Mass Spectrometry Group, University of Antwerp, Antwerp, Belgium; 2 Receptor Biology Laboratory, University of Antwerp, Antwerp, Belgium; 3 Molecular Spectroscopy Group, University of Antwerp, Antwerp, Belgium; 4 Flemish Supercomputer Centre, Antwerp, Belgium; 5 Laboratory of Medical Biochemistry, University of Antwerp, Antwerp, Belgium; 6 Astbury Centre for Structural Molecular Biology, University of Leeds, Leeds, UK; 7 School of Molecular and Cellular Biology, University of Leeds, Leeds, UK

**Keywords:** Intrinsically disordered proteins, Mass spectrometry

## Abstract

α-Synuclein is an intrinsically disordered protein that can self-aggregate and plays a major role in Parkinson’s disease (PD). Elevated levels of certain metal ions are found in protein aggregates in neurons of people suffering from PD, and environmental exposure has also been linked with neurodegeneration. Importantly, cellular interactions with metal ions, particularly Ca^2+^, have recently been reported as key for α-synuclein’s physiological function at the pre-synapse. Here we study effects of metal ion interaction with α-synuclein at the molecular level, observing changes in the conformational behaviour of monomers, with a possible link to aggregation pathways and toxicity. Using native nano-electrospray ionisation ion mobility-mass spectrometry (nESI-IM-MS), we characterize the heterogeneous interactions of alkali, alkaline earth, transition and other metal ions and their global structural effects on α-synuclein. Different binding stoichiometries found upon titration with metal ions correlate with their specific binding affinity and capacity. Subtle conformational effects seen for singly charged metals differ profoundly from binding of multiply charged ions, often leading to overall compaction of the protein depending on the preferred binding sites. This study illustrates specific effects of metal coordination, and the associated electrostatic charge patterns, on the complex structural space of the intrinsically disordered protein α-synuclein.

## Introduction

The intrinsically disordered protein (IDP) α-synuclein (α-syn) lacks a defined, unique structure in its native state and can adopt many different conformations^1^. While α-syn is predominantly present in pre-synaptic terminals in the brain, smaller amounts are found throughout the body, e.g. in blood, in the enteric nervous system in the gut, and in heart and muscle tissue^2–6^. Therefore, in vivo, the protein needs to adapt to different physiological environments where it experiences various biophysical conditions, such as pH, salt and metal ion concentrations^7–9^. These external factors can be a trigger for structural transitions and therefore potentially reshape the conformational space of α-syn^10–13^. The protein α-syn can self-associate into oligomers, condensates, protofibrils and eventually mature fibrils which occur in various structural forms^14^. Aggregates of α-syn are important components of Lewy bodies, the most common pathological characteristic of Parkinson’s disease (PD), found in dopaminergic neurons^15^. Brain regions affected by neurodegenerative diseases such as PD or Alzheimer’s disease (AD) are found to contain significantly higher concentrations of metal ions such as zinc, iron and copper^16–20^. Divalent metal ions are known to play an important role in various biological processes such as transcription regulation, signal modulation and enzyme activation^21–23^. Physiologically relevant metals such as Ca^2+^, which controls neurotransmitter release, are now proposed as important modulators of α-syn monomer conformations^8,24,25^.

While the exact physiological function of α-syn is still under debate, it is believed that it interacts with negatively charged lipids at the pre-synapse and plays a role in vesicle trafficking^8^. Metal ions can play an important role in this process, for example the charge distribution of the negatively charged C-terminal region can be profoundly affected by Ca^2+^ binding that can modulate subsequent protein-membrane interactions^8^.

It is well known that variations of the α-syn sequence, e.g. mutations and posttranslational modifications (PTMs), affect its structure and fibrillation behaviour^10,26,27^. A key factor that influences the conformational space of IDPs is the effect of charge^28–31^. The 140 amino acid long sequence of α-syn contains three separate regions (Supplementary Fig. S1). The first is the N-terminal region, amino acid (aa) 1–60, which has an overall positive charge and adopts an α-helical structure when interacting with, especially negatively charged, biological membranes. The central hydrophobic non-amyloid-β component (NAC) region (aa 61–95) is the part involved in the formation of β-sheets when protein aggregation occurs and fibrils are formed. The C-terminal region (aa 96–140) contains many negatively charged residues and is an important binding region for various metal ions^32–35^. This C-terminal region is, however, also where some of the most common PTMs on α-syn occur as well as frequent truncations. How PTMs and metal ions binding to α-syn influence each other is described in a recent review by González et al.^36^. Metal ion coordination, or protein phosphorylation, are examples of how alterations in electrostatic charge can drastically reorganize H-bonds and salt bridges at a local microstructure level. In addition, long-range effects may also affect the overall protein conformation. IDPs have been described as being more sensitive to altered charge distributions than globular, folded proteins, with charge patterns and densities suggested as key factors determining their conformational space^29^. Environmental factors such as pH, temperature, ionic strength and crowding agents can play an important role as well^37–39^.

There is however a lack of understanding how the interaction of metal ions with α-syn, and their conformational effects, might affect the formation of molecular forms which are toxic to the cell^40–42^. Structural transitions, possibly at the monomer level, are believed to be important for native protein function. But they may also cause misfolding leading to aggregation, or the formation of intermediate states (e.g. condensates) which can feed into protein aggregation pathways^43–45^. Conformational changes of α-syn monomers are hypothesized to provide a starting point for aggregation pathways that need to be better understood in order to prevent spreading of the disease throughout the brain^46,47^.

We investigate the binding affinity and capacity of a number of relevant metals and counterions and their effects on the conformational behaviour of the protein. Are conformational effects due to the specific coordination chemistry of the ions, or is there a more general charge effect when metal ions bind? Using native nano-electrospray ionisation and ion mobility-mass spectrometry (nESI(-IM)-MS) we compare the effect of binding of different numbers of mono- and multivalent metal ions with increasing concentration, including the role of counterions. Ion mobility facilitates the capacity to separate α-syn monomers based on their overall size and shape (Collision Cross Section, CCS), and thus detect multiple concurrent conformational families present in the sample.

## Results

### Conformational space of α-syn

**Figure 1:**
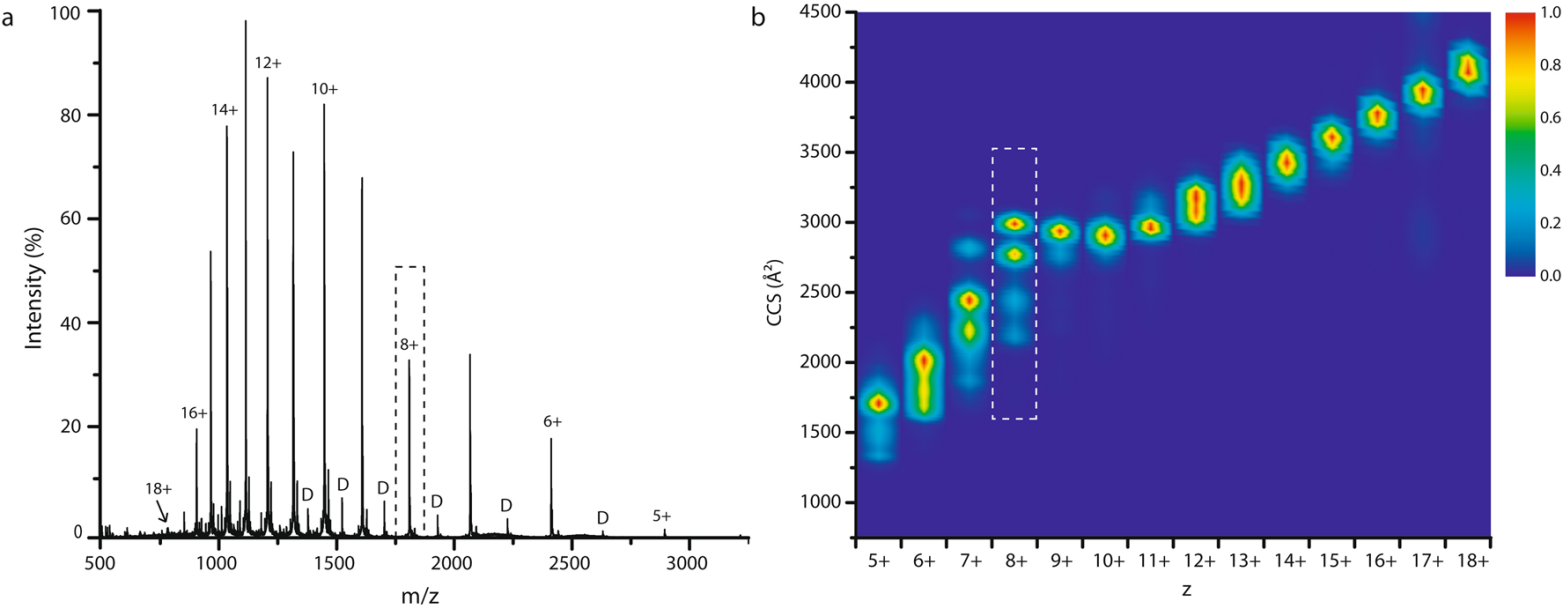
(**a**) The ESI mass spectrum of α-syn under native conditions shows a monomer charge state distribution ranging from 5+ up to 18+. Low intense dimer peaks are indicated with ‘D’. (**b**) The CCS heat map shows the populated conformational space per charge state (5+ to 18+) for α-syn monomers. The relative intensity of each CCS profile (vertical columns, per charge state) is normalised separately within a scale from 1: very intense (red) to 0: not present (dark blue), whereas the overall intensities of different charge states relative to each other are apparent in (**a**). The dashed box shows that for the 8+ state, also indicated in (**a**), four different conformational families are present.

As a natively unfolded protein, α-syn adopts both compact and extended states. Native MS is uniquely capable of capturing this diversity of molecular forms in an unbiased manner. In the ESI process, the number of proton charges found on a protein when released from the liquid droplet, is believed to correlate with the solvent accessible surface area (SASA) and therefore the conformation at that moment. Denatured and more extended forms pick up more charges, compared to native or more compact states^48,49^. The resulting nESI mass spectra of an IDP show very broad, multimodal charge state distributions. Monomer charge states range from 5+ to 18+ for α-syn (Fig. 1a), representing a spectrum of both compact and extended states.

Figure 1b represents the conformational space of α-syn monomers by the CCS distributions derived from ion mobility measurements. CCS “fingerprints” are shown for each charge state in a vertical column, as a composite heat map with colour indicating intensity (workflow Supplementary Fig. S2). Within one charge state, sometimes multiple conformational poses can be identified by their rotationally averaged size (CCS). Very compact conformations are observed for the lowest charge states, and increasingly extended conformations with larger CCS values for the most highly charged ions. The relationship is however not entirely straightforward for an IDP such as α-syn, as can be seen at intermediate charge states (7+ to 9+) which represent a conformational subspace with contributions from both compact and extended states. A comparison with CCS values calculated from nuclear magnetic resonance (NMR) spectroscopy ensembles in combination with conformational restraints derived from small angle X-ray scattering (SAXS) (Protein Ensemble Database 9AAC) is in general accord with our ion mobility data in the 7+ to 14+ range^50,51^. This is an important indication that CCS values from gas phase structures, obtained under gentle energetic IM-MS conditions, correlate well with those of α-syn in solution. The intermediate 8+ ion will be used here as a representative state in order to monitor shifts in the conformational ensemble upon metal ion binding. The 8+ charge state demonstrates a characteristic pattern with four prominent conformational families in the CCS range of 2200–3000 Å^2^, which have been described before^52^.

### Binding affinity and capacity of α-syn for different metal ions

**Figure 2:**
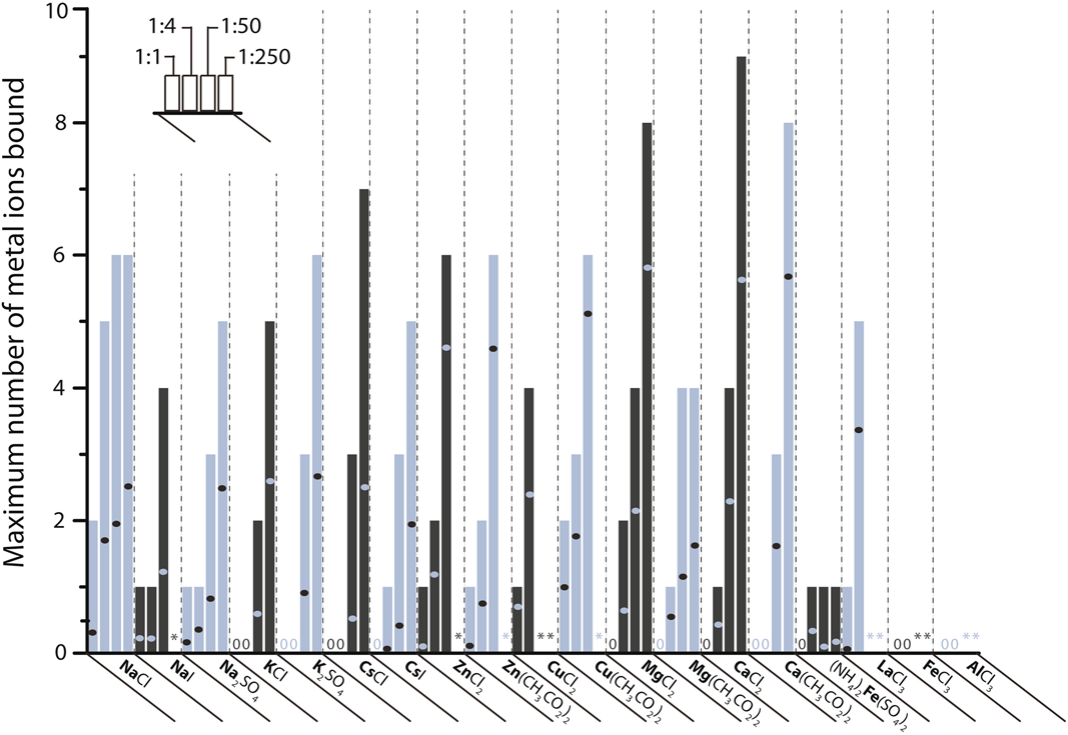
Maximum and average number of binding of various metal ions (in bold) to the 8+ charge state of α-syn. Per metal salt four bars are shown which correspond to protein:metal ion ratios of 1:1, 1:4, 1:50 and 1:250 from left to right. The height of each bar indicates the maximum number of metal ions bound (S/N ≥ 3), and the dot in each bar corresponds to the average metal binding (weighted peak intensities of all stoichiometries detected, including the unbound state when present). In cases where it was difficult to determine the stoichiometry due to poorly resolved signal, an asterisk* is shown. When no binding of metal ions was observed, ‘0′ is shown.

Next, we studied the extent of interaction with a range of different metals, which have physiological roles or are believed to participate in protein aggregation, using native nESI-MS^53–55^. Metal ion binding is observed across the whole charge state spectrum fairly evenly (not shown here), but we highlight the 8+ ions again. The maximum and average numbers of ions bound to α-syn monomers are summarized in Fig. 2 for various metals with valences from 1+ to 3+, at increasing excess of metal ions. The metal ion concentrations used here cover a broad range from 20 µM (protein:metal ratio 1:1) representing physiologically relevant levels, to 5 mM (1:250), giving an indication of binding capacity. As an example, in neuronal cells Ca^2+^ concentrations can fluctuate between 50 nM and 1 µM when certain cellular processes are activated^56^.

At the equimolar 1:1 ratio (left bar in each section in Fig. 2), Na^+^, Zn^2+^, Cu^2+^, and La^3+^ show binding above the significance threshold chosen here (signal to noise ratio, S/N ≥ 3), indicating a high relative affinity of α-syn for those metals. Supplementary Fig. S3 details the percentage of unbound protein per metal salt present at four different ratios, and again Cu^2+^, Na^+^, Zn^2+^, and La^3+^ appear as high affinity binders (in that order) with the least unbound protein at a 1:1 ratio. K^+^ only binds at 50-fold excess or more, indicating a dramatically lower affinity. This preference for Na^+^ over other alkali metal ions is in fact a well-known property of proteins; ions with similar hydration free energies are expected to form the most stable ion pairs, and Na^+^ and carboxylate groups are well matched^57,58^. In line with that, the type of counterion also plays a role for Na^+^, but less so for K^+^ and Cs^+^ interactions with α-syn. The role of counterions will be discussed further below.

Among the 2+ ions, Zn^2+^ and Cu^2+^ show high affinity for stoichiometric binding (1:1 ratio). Fe^2+^ is only interacting weakly with one ion bound at 1:4 protein:metal ratio and higher. Mg^2+^ and Ca^2+^ also bind α-syn only at 1:4 ratios and above. Interestingly, there is a remarkable binding capacity for Mg^2+^ and Ca^2+^ with coordination of up to eight and nine metal ions, respectively. This observation indicates that binding affinity and binding capacity are not linked in a straightforward manner with each other. Low affinity binding ions such as Mg^2+^ and Ca^2+^ also tend to demonstrate a very high binding capacity, i.e. the K_d_ values for additional binding remain at a similar level. The opposite is seen, i.e. a lower overall binding capacity, for high affinity ions such as Na^+^. Figure 2 also shows the average number, calculated from the intensity-weighed stoichiometries, of metal ions bound per salt and ratio, as dots within the bars. For a given ratio, fewer 1+ metal ions bind to α-syn than multiply charged metal ions. Trends for the average and maximum number of ions binding match broadly, which is seen as a result of the Gaussian-like distribution of bound states without any distinct preferences for a specific number of metal interactions (see below). Coordination of multiply charged metal ions, albeit at lower affinity, appears to be a characteristic of α-syn with its overall negatively charged sequence. Known K_d_ values in literature for binding of metal ions to α-syn span a wide range 0.2 nM–60 µM for Cu^2+^, 72 µM and 52 µM for Fe^2+^ and Fe^3+^, respectively, and around 21 µM for Ca^2+^^8,10,59^. Our results for these ions, except for Fe^3+^ where only clustering was observed, correlate quite well with these K_d_ values from literature, based on the ratios where binding of these ions was detected (Fig. 2). No representative mass spectra could be recorded for Zn^2+^ and Cu^2+^ at high ratios (1:250), as the signals were of insufficient quality to define binding stoichiometries, most likely due to interference with metal salt clusters or colloids formed at these high concentrations. The 3+ metal ions studied here have also yielded poor quality spectra at 1:50 and higher metal ratios, preventing further analysis. For lower metal ion concentrations only La^3+^ is seen to bind to α-syn, while binding of Al^3+^ and Fe^3+^ appeared too weak to be detected.

**Figure 3:**
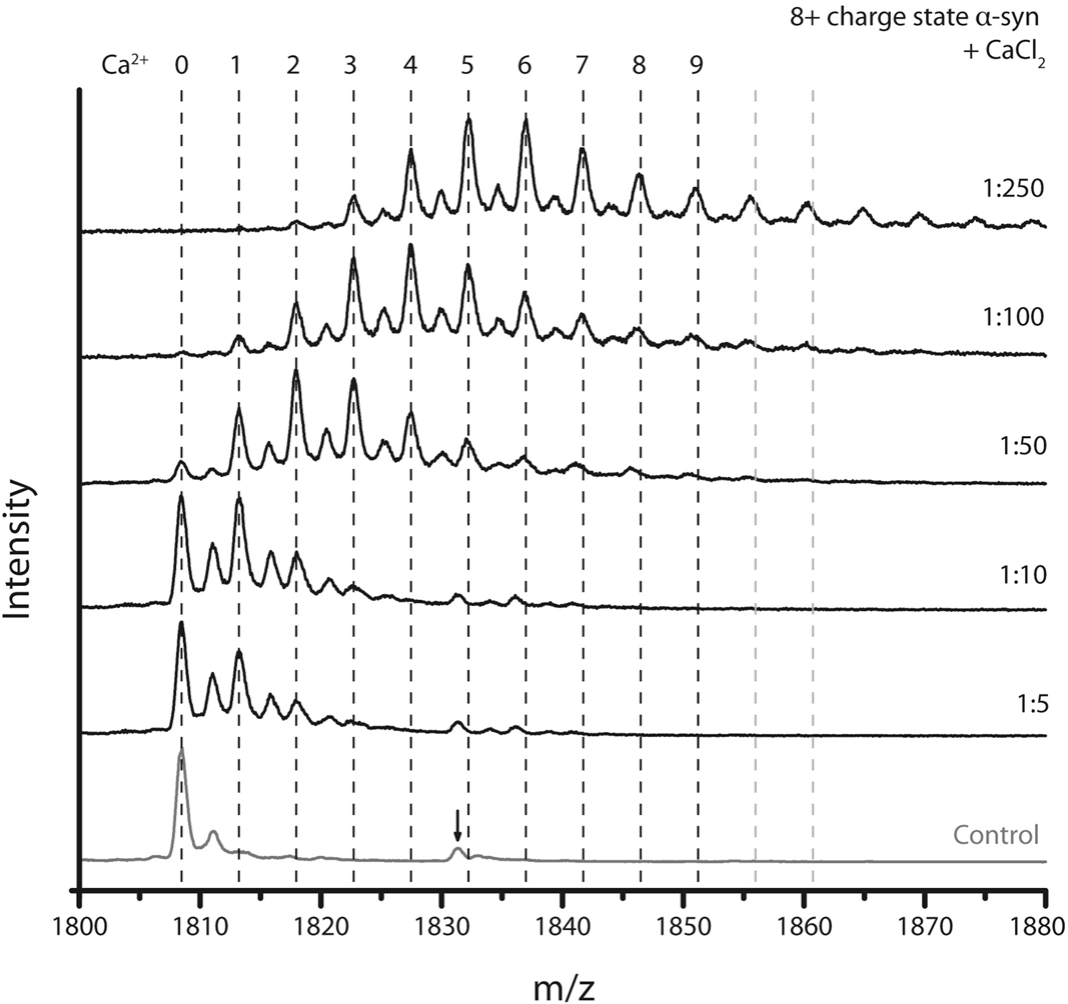
Mass spectra of the titration of α-syn with CaCl_2_. The dashed lines indicate the theoretical m/z values of the 8+ charge state of α-syn with a specific number of Ca^2+^ ions bound, at the top of the dotted line. The control shows only the unbound peak, indicated by ‘0’ and with one Na^+^ adduct still remaining after thorough purification steps, next to a contaminant (peak indicated with an arrow), which also seems to bind Ca^2+^ with a similar peak distribution as α-syn itself. With increasing CaCl_2_ concentration more Ca^2+^ ions bind with a maximum of nine, defined by a S/N ≥ 3 of the peak at the correct m/z value. Grey dotted lines indicate theoretical values of the 8+ charge state of α-syn with more than nine Ca^2+^ ions bound and do not align with the peaks in the spectrum, which can be attributed to a lower number of Ca^2+^ bound to the contaminant.

Figure 3 exemplifies Ca^2+^ binding further with a stepwise CaCl_2_ titration. The distribution of different Ca^2+^-bound states appears smooth, without evidence for “magic numbers” of binding sites or cooperativity. Thus, metal coordination appears to be concentration-dependent and proceeds in a gradual manner. Earlier research using mass spectrometry already reported the binding of up to six Ca^2+^ ions to α-syn at a 1:360 protein to metal ion ratio. Data from ^1^H–^15^N HSQC NMR spectra have previously suggested the effective binding of up to four Ca^2+^ ions^8^. Our results show a maximum number of nine Ca^2+^ ions binding to α-syn, indicating that the binding capacity of α-syn towards this metal ion is indeed very high. It might seem in Fig. 3 that even more than nine Ca^2+^ ions are binding at a 1:250 ratio (dotted lines indicate theoretical m/z values for higher binding stoichiometries); however, those peaks do not correspond entirely with the calculated m/z values for Ca^2+^ stoichiometries and are likely due to other impurities.

As a point of note, coordination of up to nine divalent Ca^2+^ ions equates to a nominal addition of 18 positive charges. No significant shift in the overall charge state distribution was however detected for any of the metal ions, except La^3+^ where a slight intensity shift towards lower charge states might indicate that compaction is significant enough to see an effect on the charge state distribution (Supplementary Fig. S4). The 8+ charge state requires the presence of ten negative charges in order to achieve a net charge of 8+ when nine Ca^2+^ are bound, also written as [M + 9 Ca^2+^–10 H^+^]^8+^. If these negative charges were additional ligands (counterions), the additional mass would also show up in the adduct spectra, but this is not the case here. The pI of α-syn is 4.67 and the protein contains 24 negatively charged residues at neutral pH, attributable to aspartic acid and glutamic acid, and 15 positively charged lysine residues. It is therefore conceivable that deprotonated carboxyl groups, which are also good coordination sites for Ca^2+^ ions, provide the additional negative charges.

### Conformational effects of metal ion binding

Having established that many types of metal ions interact with α-syn, albeit with different affinities and stoichiometries, we now set out to study if, and how, they change the protein’s conformational space using ion mobility. We aim to understand if observed structural effects are specifically caused by the binding of certain metals triggering conformational change by nature of their coordination, or if structural changes are more generally related to an altered charge “landscape” across the protein following metal ion interaction. Electrostatic effects play an important role in protein structure, and metal ion binding is no exception; not just through the formation of intramolecular ion pairs, but long-range Coulomb effects and changes in local charge density can be major triggers for conformational change or even order–disorder transitions^55,60^.

**Figure 4:**
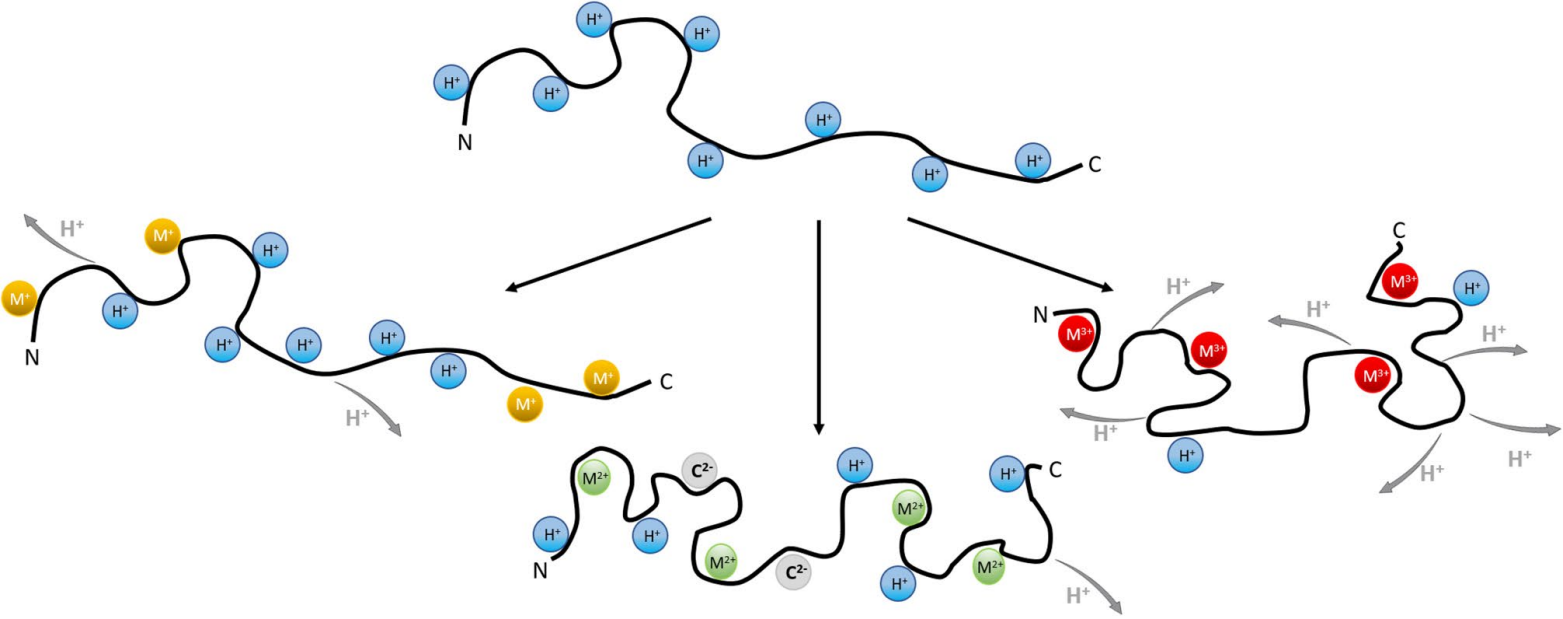
Changing the charge distribution of a protonated IDP using singly or multiply charged metal ions to replace one or several proton charges while maintaining the original 8+ ESI charge state. Singly charged ions (yellow) replace protons (blue), while each doubly charged ion (green) replaces two and each triply charged ion (red) three protons. Zwitterionic states are expected with deprotonated and additional protonated sites, and deprotonation and counterions (C^2−^, in grey) can account for the net charge of 8+.

The protons α-syn obtains during electrospray ionisation are known to distribute evenly across the protein surface due to Coulomb repulsion. H^+^ ions remain relatively mobile post-ionization and can locate at many different sites of solution or gas-phase basicity (Fig. 4 top)^61^. It is important to reiterate that the number of charges a protein attains during the ESI process depends on its exposed surface in solution, and protonation itself does not result in conformational changes. Metal ions on the other hand may well have structural effects when binding to the protein in solution, possibly altering its conformational behaviour. Metal ion coordination is expected to be specific for certain amino acids or binding sites. Bi- and trivalent ions can also cause significant shifts in local charge density, affecting protein structure. While this is true for all metal ion-protein interactions, IDPs in particular due to their lack of defined and stable 3D structure may well show significant structural effects upon metal ion coordination.

The observation that no significant shifts are detected in the ESI charge state distribution upon metal ion binding (with the exception of La^3+^, see above; only slight intensity shifts as seen in Supplementary Fig. S4) would indicate that no large-scale compaction or extension of the structure occurs. This however does not preclude more subtle changes that can be detected by ion mobility. Here we monitor shifts in the drift time or CCS distributions of the 8+ α-syn monomer charge state upon metal binding. We can extract such CCS data for each metal bound state individually from the corresponding peaks in the mass spectra (workflow described in Supplementary Fig. S1).

**Figure 5:**
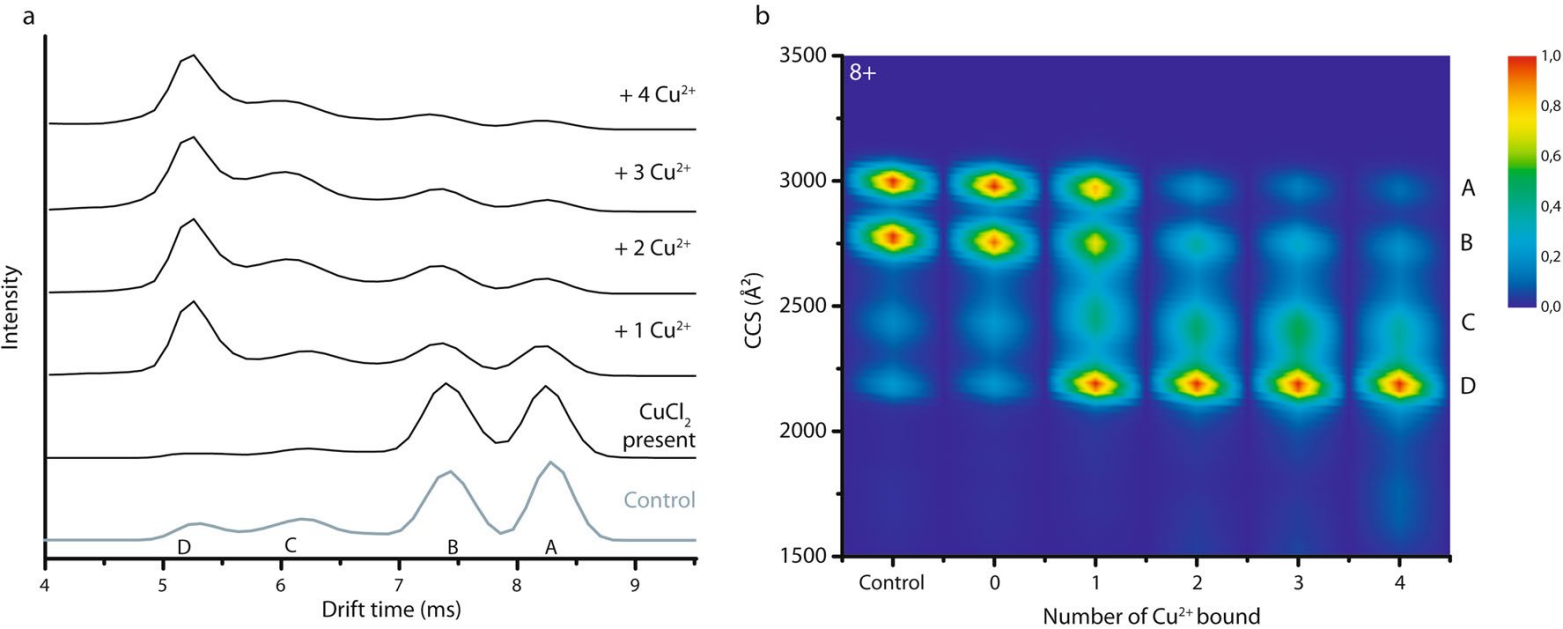
(**a**) Drift time profile for the 8+ charge state of α-syn with different numbers of Cu^2+^ bound. The control shows the conformations of α-syn without metal ions present in the sample. (**b**) Drift times are converted to CCS plots after calibration. ‘0’ indicates the presence of CuCl_2_ but shows the unbound state. Different conformations are labelled “A” (most extended) to “D” (most compact). Intensity scale as in Fig. 1b.

Figure 5a displays drift time plots of the conformational ensemble of the 8+ ions when CuCl_2_ is present in the sample. The heat map shown in Fig. 5b combines data from IM-MS measurements of α-syn in the presence of CuCl_2_ at 1:1 and 1:4 protein:metal ion ratios, in order to achieve stoichiometries from zero up to four ions bound. The “Control” CCS plot is obtained from the mass spectrum where no CuCl_2_ is present in solution, while “0” indicates CuCl_2_ is present in solution, but the CCS profile is extracted from the m/z of the unbound 8+ peak. Conformational families are labelled “A”–“D” in the same way as the drift plots, with “A” as the most extended conformation and “D” the most compact one. The intensity shifts from peaks “A” and “B” gradually and almost completely to peak “D” with increasing Cu^2+^ concentration. An earlier study already indicated a compacting effect when Cu^2+^ binds to α-syn^62^, but here the gradual compaction observed indicates that each additional metal ion bound alters the conformational ensemble of the α-syn monomers further. This would mean that not only the first binding site, or the first ion that binds, alters the α-syn monomer conformation and intramolecular interactions, but the effect is further enhanced with additional metal coordination. This experiment also provides an important control to prove that metal ions that bind to the protein in solution are not lost in the gas phase during electrospray ionization or analysis inside the instrument. If this were to occur, the ion mobility signal of the metal-bound states would contribute to the detected metal-free form (“0 bound”) via a kind of conformational “memory effect”. Instead these gas-phase experiments reflecting metal ion interactions with the protein and conformation effects in the condensed phase.

As explained above and detailed in Fig. 1, the 8+ charge state provides a very useful “fingerprint” of the complex behaviour of the protein. The width and heterogeneity of the drift time peaks might indicate that more than one conformation contributes to each peak. Either several, very similar, conformations are present at the same time, or there is a dynamic exchange occurring between different, yet very similar, structural states that causes peak broadening, changes which are too dynamic to be detected individually using IM-MS.

Supplementary Fig. S4 shows that metal ion binding is detected at other intermediate charge states (7+ and 9+) as well, and Supplementary Fig. S5 shows the gradual compaction upon binding increasing numbers of Zn^2+^. In contrast to this behaviour, CCS plots of lysozyme, which has a molecular weight comparable to that of α-syn but a fixed globular 3D structure, demonstrate that there is no conformational effect when Zn^2+^ binds (Supplementary Fig. S5), a finding confirmed with other metal ions (similar results as in Supplementary Fig. S5).

**Figure 6:**
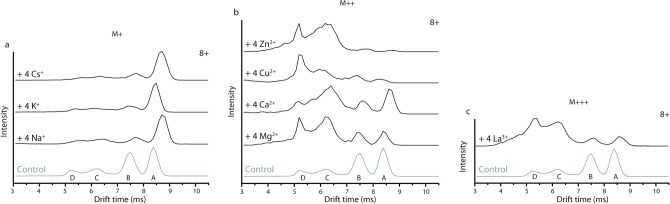
Comparison of drift time profiles representing conformational ensembles for the 8+ charge state of unbound α syn (control) and with four metal ions bound, arranged per oxidation state. (**a**) Singly charged alkali metal ions (M+) shift the distribution towards the most extended conformation “A”. (**b**) Doubly charged metal ions (M++) have the opposite effect with compact states (“C” and “D”) dominating, but the drift time profiles look different in each case. (**c**) La^3+^ binding (M+++) leads to compaction similar to doubly charged ions, but again the conformational pattern is different. Different conformations are labelled “A” (most extended) to “D” (most compact).

In Fig. 6 we compare drift time profiles for 8+ α-syn with different metal ions bound, grouped according to oxidation state, with the 8+ protonated (i.e. unbound) form as reference. The four metal ion bound state is shown and Cl^-^ salts were used. When monovalent ions such as Na^+^, K^+^ and Cs^+^ bind to α-syn (Fig. 6a) the most extended conformation labelled as “A” becomes dominant, with little difference between the alkali metals. This is also true for different binding stoichiometries (Supplementary Fig. S6) which demonstrates essentially the same CCS profiles: the intensity shifts away from the more compact conformations “B”, “C” and “D” to the extended conformation “A”. Singly charged alkali metal ions are more likely to bind α-syn at specific sites compared to protons, likely interacting with negatively charged side chains of aspartic and glutamic acid residues and possibly also carbonyl groups of the protein backbone^63^. When negative charge sites (e.g. carboxylates) in the protein are neutralised by binding 1+ metal ions, their ability to form salt bridges with other parts of the protein sequence, and corresponding residual compactness, are weakened. Overall charge reduction in the C-terminal region upon metal binding thus results in locally, and potentially globally, altered electrostatic interactions. Unlike bi- or trivalent ions, alkali metal ions are typically not able to coordinate different, remote parts of the protein sequence in their ligand sphere.

Drift time profiles are also shown for the 8+ charge state of α-syn with four of the doubly charged ions Mg^2+^, Ca^2+^, Cu^2+^ and Zn^2+^ (Fig. 6b) and with four La^3+^ ions bound (Fig. 6c), respectively, alongside the same unbound control. In contrast to the singly charged ions, the compact states (peaks “C” and “D”) in the conformational ensemble are now more substantially populated, indicating binding of multiply charged metal ion induces compaction of α-syn monomers. The form with four metal ions bound is shown here to represent conformational changes, but as seen for Cu^2+^ in Fig. 4 and Zn^2+^ in Supplementary Fig. S5 gradual compaction occurs with every additional metal ion bound. Circular dichroism spectroscopy experiments confirm that compaction of the tertiary protein structure, here represented by CCS values, occurs without the formation of secondary structure as shown in Supplementary Fig. S7.

When α-syn binds multiply charged metal ions, the charge site distribution in the protein changes even with the overall ESI charge state maintained. This results in fewer and more specific charge sites, affecting the overall electrostatics along the protein sequence, and through space. The observed general compacting trend is in line with the hypothesis that Mg^2+^ and Ca^2+^, and particularly Cu^2+^ and Zn^2+^, coordinate more than one group or amino acid in their ligand sphere, causing a local or even more far-reaching structural reorganization, and therefore overall more compact states (Fig. 4 middle and right). Supplementary Fig. S8 summarizes these effects in CCS heat maps. Notably the drift time profiles of multivalent metal ion-bound states differ considerably between the metals, indicating that the conformational compaction of the monomer is not just simply an electrostatic effect. Rather, these metal ions remodel the conformational behaviour of α-syn in a specific fashion. Their ionic radii and specific coordination chemistries predispose them towards certain binding sites, and their ability to coordinate with different residues allows them to reorganize the protein chain both locally and globally. It is important to note that the observed drift time and CCS profile for a specific number of metal ions bound does not depend on the protein:metal ion ratio used to prepare it (Supplementary Fig. S9).

### Counterions

**Figure 7:**
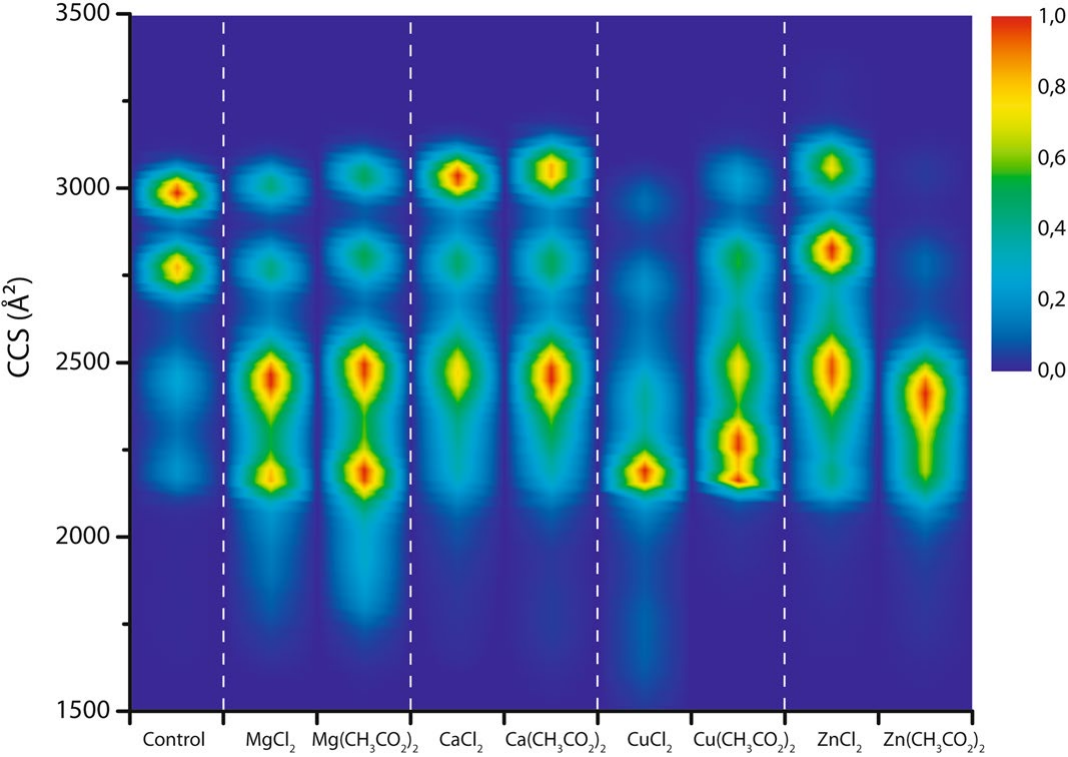
CCS profiles comparing conformational ensembles for the 8+ charge state of α syn without any (control) and with four Mg^2+^, Ca^2+^, Cu^2+^ or Zn^2+^ ions bound, either added as chloride or acetate salts in a 1:50 protein:metal ion ratio. All metal salts lead to compaction as also seen in Fig. 6, yet the compaction pattern is characteristic for every metal salt indicating that the counterions plays an important role, in spite of not being detected as bound to the protein. Intensity scale as in Fig. 1b.

In addition to conformational effects related to the binding of metal ions to α-syn, we also investigated the role of specific counterions. Counterions that are not detected bound to α-syn in the mass spectra still seem to have a small effect on the binding affinity (Fig. 2), and a sometimes more significant effect on the conformational profiles of the associated metal ion-bound states. This is likely due to their volatility; they can easily evaporate in their protonated form leaving a deprotonation site behind. In Fig. 7 CCS profiles of the 8+ α-syn monomer with four metal ions (Mg^2+^, Ca^2+^, Cu^2+^ and Zn^2+^) bound are compared according to whether chloride or acetate salts were used. Importantly, the metal ions studied all induce compacting effects of α-syn monomers upon binding, as seen before in the drift time profiles in Fig. 6b, irrespective of the counterions. The actual compaction pattern however can depend somewhat on the type of salt, for a specific metal ion and stoichiometry.

Counterions can act as ligands for multiply charged metal ions and may therefore affect their coordination with the protein, potentially playing a role in directing metal ions to different binding sites or regions and affecting the conformations, even in cases where the counterions are lost during the ESI process. Supplementary Fig. S10 shows these effects can be observed with one to three metal ions bound too. The differences for the chloride vs. acetate salts of Mg^2+^ and especially Ca^2+^ are less profound than the effect on CCS patterns for Cu^2+^ and Zn^2+^, with their more specific coordination chemistry and more drastic compacting effects on α-syn (Fig. 6). The protein conformation is very sensitive to the binding of metals such as copper and zinc, and their precise modes and sites of coordination, which are also controlled by anionic ligands.

**Figure 8:**
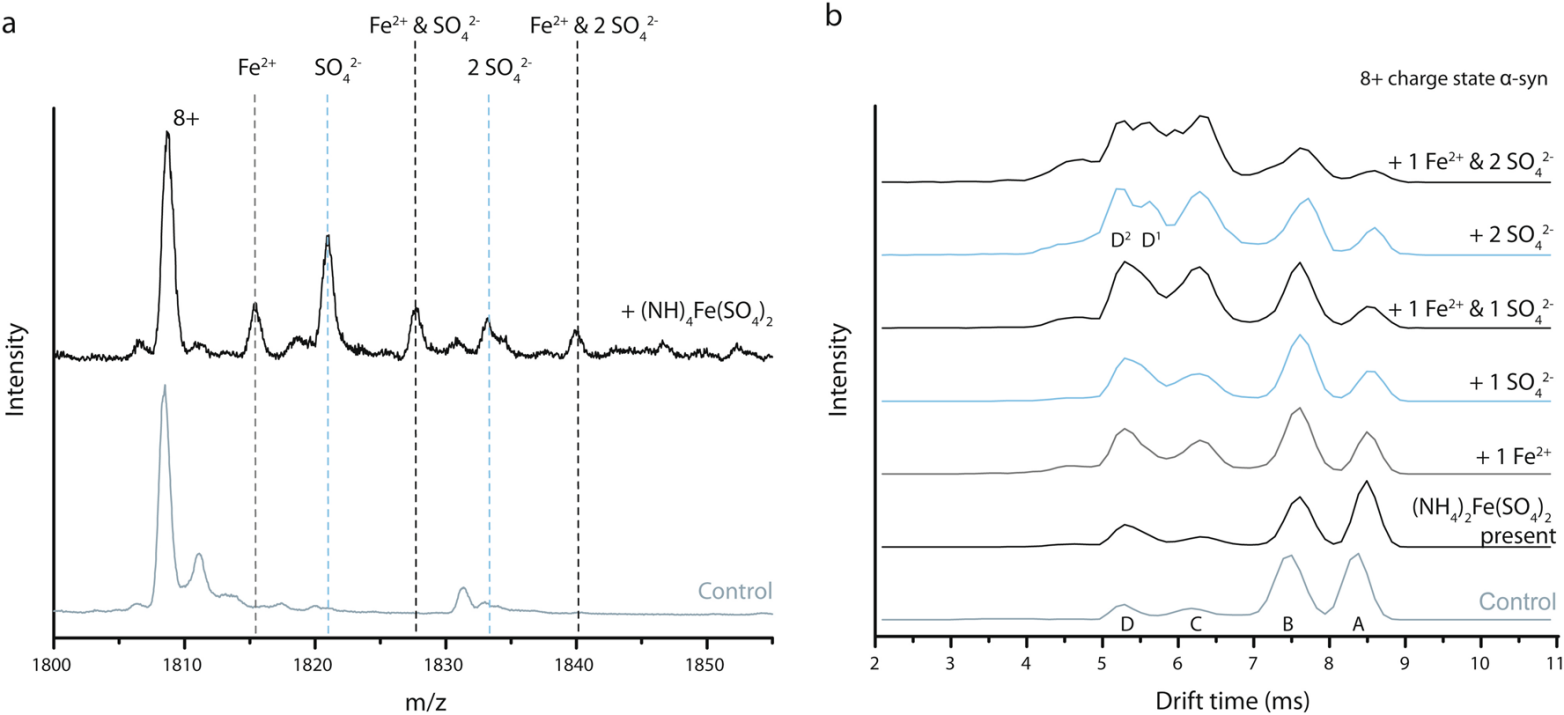
(**a**) Mass spectrum of the 8+ charge state of α syn without ions bound, in absence (control) and presence of 5 mM (NH_4_)_2_Fe(SO_4_)_2_. Binding of Fe^2+^ is indicated in grey while SO_4_^2−^ binding is indicated in blue. Black dashed lines indicate a peak with one or two SO_4_^2−^ bound and one Fe^2+^ bound. (**b**) Comparison of drift time plots of the 8+ charge state of α syn in the absence (Control) and presence of 5 mM (NH_4_)_2_Fe(SO_4_)_2_ resulting in various bound states. Different conformations are labelled “A” (most extended) to “D” or “D^1^/D^2^” respectively (most compact).

Unlike for Cl^−^, I^−^ and acetate, which are not retained in the mass spectra due to being volatile in their protonated form, binding of sulfate groups is detected by the additional mass. Sulfate binds to α-syn when (NH_4_)_2_Fe(SO_4_)_2_ is present in a 1:250 α-syn to salt ratio. Figure 8a shows that under those conditions, sulfate can bind to the protein twice while the associated metal ion, Fe^2+^, binds once. It comes as no surprise that SO_4_^2−^ has the ability to associate with proteins, considering ammonium sulfate is often used in a precipitation step as part of a protein purification protocol. For Na_2_SO_4_ and K_2_SO_4_ however, no binding of SO_4_^2−^ to α-syn is detected (data not shown), suggesting that these salts dissociate fully and only the alkali metal ions interact with the protein. Clearly the combination of metal and counterion is important here, and sulfates interact more strongly with multivalent ions which brings them both within the association sphere of the protein and causes them to induce conformational effects.

The drift time profile of the 8+ charge state of α-syn in presence of (NH_4_)_2_Fe(SO_4_)_2_ (no binding) is nearly identical to the control (Fig. 8b), which confirms that neither ions are lost during the ESI process (not being volatile). With one SO_4_^2−^ ion binding, the distribution shifts towards more compact protein conformations. When two SO_4_^2−^ ions are bound, the intensity of peaks “C” and “D” increase even more, with peak “D” apparently splitting into two peaks “D1” and “D2”. In summary, bivalent sulfate ions have a compacting effect roughly comparable to 2+ metal ions. This effect of sulfate ions can be explained in the same way as for cations, altering local charge density on the protein possibly by interacting with the positively charged N-terminal region and lysine residues, hereby affecting intramolecular electrostatic interactions and conformational behaviour. When Fe^2+^ and SO_4_^2−^ both bind to α-syn, an almost additive compacting effect can be detected compared to binding of either ion separately. Since Fe^2+^ and SO_4_^2−^ are likely to have very different binding preferences, both ions can alter electrostatic interactions in separate regions of the protein in similar ways.

## Discussion

In neurons α-syn makes up around 1% of total protein in the cytosol^64^. In cells and also in the extracellular space, concentrations of up to 100 µM can be reached^65^. Since nESI-IM-MS however works best at lower protein concentration (low µM range) avoiding ion clustering and increased noise levels, experiments in this study were performed on samples with a final concentration of 20 µM α-syn. In vivo local α-syn concentrations may fluctuate drastically, and crowding effects inside the cell must also be taken into account. While biophysical experiments cannot easily reproduce all aspects of the in vivo scenario, there is also little consensus in the literature about levels of specific metal ions in neurons, and the effective metal ion concentration depends also on the presence of other proteins which may interact with them. The intraneuronal concentration of Na^+^ and K^+^ can be as high as 18 mM and 135 mM respectively, maintaining cell homeostasis^66^. For doubly charged ions concentrations of up to 150 µM for Zn^2+^ and even 250 µM for Cu^2+^ are found^67,68^. The protein:metal ion ratios used in this study cover some of the physiological range (1:1 and 1:4 ratio), but also challenge the protein at higher concentrations with regard to binding capacity and further possible conformational changes.

Previous research has reported various metal ion binding regions in α-syn; especially in the highly negatively charged C-terminal domain^8,69,70^. The exceptionally high binding capacity for Ca^2+^ is likely related to α-syn’s physiological role in the pre-synaptic space where intracellular calcium management is vital for neurotransmitter vesicle release^8^. There remains however an important knowledge gap about the structural effects of metal ion interactions with α-syn more broadly, and molecular mechanisms that define conformational states, oligomerization and gel-like condensation behaviour, as well as factors that can subsequently lead to various aggregation pathways.

A key question here arises as to where exactly the different types of metal ions bind: are there many available low-affinity sites, or does binding occur mostly clustered in one or a few specific regions? Do the binding sites and therefore the charge locations define the conformational behaviour of the IDP, or is the specific coordination chemistry of the ions important? If there were any high affinity sites, this would become apparent in the binding pattern at increasing metal ion ratio; however all of the metal ions studied here as well as the sulfate counterions show a gradual increase with concentration rather than any evidence for preferred stoichiometries of binding.

The compacting trends observed here were also observed in earlier studies, confirming our nESI-IM-MS results^7,45^. Alkali metal ions are known to interact almost exclusively with oxygen atoms in proteins, with a single negative charge as “ligand” (besides additional water molecules which are not retained in ESI mass spectra)^63^. Obvious binding partners are deprotonated aspartic and glutamic acid side chains, but also hydroxyl groups and especially backbone carbonyl oxygen atoms^71^. Multiply charged ions are more diverse: Mg^2+^ and Ca^2+^ behave somewhat like alkali metal ions, since they also prefer interacting with oxygen atoms. Due to their 2+ charge they will mainly interact with carboxylates in Asp and Glu, or sometimes with amide group oxygen atoms. Other doubly charged ions however (e.g. transition metals) are known to interact with nitrogen and sometimes sulfur atoms as well. Since α-syn does not contain cysteine, only the four methionines might play a role here, next to nitrogen containing amino acids such as e.g. histidine^71,72^. The clear difference between Mg^2+^ and Ca^2+^ on one hand and other multiply charged metal ions on the other goes some way to explain why we see stark differences in terms of binding affinity and capacity, and significant but distinct conformational effects.

The fact that many different possible interaction sites can be accessed for metal ion binding is supportive of the diversity of individual contributions to the complex conformational fingerprints which we see.

Two types of counterions were studied, “volatile” ones whose presence has an effect on protein conformation, but which evaporate during the ESI process as e.g. neutral HCl and acetic acid, and non-volatile sulfate ions that are detected bound to α-syn causing compaction similar to some doubly charged metal ions. It remains somewhat unclear however if these anions have a direct structural effect on the protein, or if they indirectly affect it via their interactions with metal ions or with the surrounding water. The hydration sphere around a protein is important for its folding and stability, and we expect that such interface effects will also play a role for the conformational behaviour of IDPs^73,74^.

Having characterized global conformational behaviour using ion mobility here, there is an obvious desire to also fully map out all the possible metal ion binding sites and their occupancy. Computational tools to predict binding sites have been proven to be very valuable tools in developing molecular models to visualise the conformations of α-syn metal ion complexes^75–79^. Using the IonCom software with various metal ions as well as counterions to α-syn, different binding sites of for example Ca^2+^, Mg^2+^, Zn^2+^ and Fe^3+^ were identified^75^. However, no binding sites were found for Na^+^, K^+^, Cu^2+^, Fe^2+^ and SO_4_^2−^ while we clearly saw these ions bound using MS. This illustrates the difficulty of predicting protein-metal ion interactions, particularly for a protein with an undefined structure and the need for more insight into these molecular mechanisms. There are also promising top-down MS/MS fragmentation approaches which can identify binding sites of metal ions in proteins, namely electron transfer dissociation (ETD), electron capture dissociation (ECD) and ultraviolet photodissociation (UVPD), but the challenge here is that the fragment-level binding data should be linked with a specific conformational state of the protein^45,80^.

Here we elucidated the specific conformational effects of metal ions on α-syn, showing the relevance and importance of this class of structural modulators which are present in vivo. Future work will try to elucidate the binding patterns of metal ions in order to fully understand how resulting conformational ensembles are formed, so that specific metal ion binding can be linked to specific structural and functional effects. As α-syn is often modified in vivo (e.g., *N*-acetylated, truncated, phosphorylated), studies of metal ion interactions and conformational effects should also be extended to these isoforms.

## Conclusion

This work highlights the capability of native nESI-IM-MS to capture the heterogeneous conformational space of biologically relevant targets that are challenging to study with more conventional biophysical techniques. The mass spectrometry approach is able to detect binding stoichiometries of a variety of metal ions and their affinity for the intrinsically disordered protein α-syn, and elucidate the effect these interactors have on the conformational space of the protein, depending on their charge but also more specifically on their chemical nature.

When altering the electrostatic balance of the protein by interaction with multiply-charged metal ions, which replace protons acquired in ESI-MS while retaining the overall charge state, there is a clear intensity shift towards more compact conformations. The observed, complex patterns are different upon binding of various metal ions, which confirms that they are specific to these ions and not just due to the mere presence of charges on the protein. However, neither compaction nor elongation of α-syn monomers alone are sufficient descriptors to make statements about the rest of the aggregation pathway in terms of oligomerisation/fibrillation or toxicity, without additional knowledge of the detailed structure of the species involved^44,81^. When singly charged metal ions bind, there is an intensity shift towards the most extended conformation. For multiply charged metal ions, the compaction pattern depends on the oxidation state of the metal ion, the binding stoichiometry, the counterion present and the chemical nature (ligand preference) of the metal ion itself. The observation of different, highly specific conformational and compacting patterns upon binding of multiply charged metal ions illustrates that nESI-IM-MS is a powerful technique in capturing detailed differences in the dynamic conformational space of α-syn.

An in-depth interpretation of these changes is of course very desirable, but currently beyond the capabilities of computational approaches. It is likely that specific metal ion binding plays an important role in how the conformational space of α-syn is affected and its biological (dys)function regulated, leading to various physiological and pathological outcomes. In order to fully capture events that can alter the aggregation pathway of α-syn and other proteins or peptides involved in neurodegenerative diseases, it will be important to further exploit the potential of MS methods for structural studies of metal ion interactions with sequence variants, oligomers, condensates and (proto-)fibrils, as well as mapping key interaction sites. This understanding will enable us to uncover the roles that metal ions may play in the structural landscape of these relevant proteins, and how they can then lead to an altered aggregation pathway and toxicity.

## Methods

### Materials and sample preparation

NaCl, KCl, ZnCl_2_, MgCl_2_·6H_2_O, FeCl_3_·6H_2_O and AlCl_3_·6H_2_O were purchased from Thermo Fisher Scientific (Waltham, Massachusetts, USA) and NaI, Na_2_SO_4_, CsCl, CsI, Zn(CH_3_CO_2_)_2_, CuCl_2_·2H_2_O, Cu(CH_3_CO_2_)_2_, Mg(CH_3_CO_2_)_2_·4H_2_O, (NH_4_)_2_Fe(SO_4_)_2_·6H_2_O, CaCl_2_·2H_2_O, Ca(CH_3_CO_2_)_2_·xH_2_O and LaCl_3_·xH_2_O from Sigma-Aldrich (St. Louis, Missouri, USA). K_2_SO_4_ was purchased from Merck (Darmstadt, Germany). All metal ion salts have a purity of 99% or higher. Lysozyme from hen egg white was purchased from Sigma-Aldrich (St. Louis, Missouri, USA).

Human wild-type α-syn was expressed and purified as described previously^82^. An additional gel filtration step was included before anion exchange. Finally, the purified protein was dialysed against 20 mM ammonium bicarbonate (Sigma-Aldrich, St. Louis, Missouri, USA) and lyophilised. For MS experiments, α-syn was redissolved in 20 mM ammonium acetate (Sigma-Aldrich, St. Louis, Missouri, USA) solution at pH 6.8 to a stock concentration of 70 µM. Metal salts were prepared as 20 mM stock solutions. In final analytical samples an α-syn concentration of 20 µM was used in the presence of 20 µM, 80 µM, 1 mM or 5 mM of metal ions. The pH was monitored upon addition of metal salt and did not change by more than 0.1. For the samples with a final metal ion concentration of 20 µM or 80 µM, the 20 mM metal salt solution was first diluted to 2 mM in deionised H_2_O. Samples were incubated for 10 min at room temperature after addition of the specific metal salt before performing the measurements.

### Nano-ESI ion mobility mass spectrometry

Native MS experiments we performed on a Synapt G2 High Definition mass spectrometer (Waters Corporation, Wilmslow, UK) with travelling wave (T-wave) ion mobility and equipped with a nanoESI source, using *in-house* generated gold-coated borosilicate capillaries. The most important experimental parameters were set at: capillary voltage 1.4–1.8 kV; source temperature 30 °C; sample cone 40 V; extraction cone 1 V; trap collision energy 4 V; transfer collision energy 0 V; trap bias 45 V; IM wave velocity 300 m/s; IM wave height 35 V. Gas pressures in the instrument were: source 2.7 mbar; trap cell 0.023 mbar; IM cell 3.0 mbar; transfer cell 0.025 mbar. Different instrument tuning (voltages and pressures) can somewhat affect the drift time patterns, however the settings remained unchanged for all experiments reported here, thereby ensuring that they are comparable across different experiments..

### Data analysis

Calibration of travelling wave ion mobility measurements was carried out based on literature collision cross section (CCS) values using denatured cytochrome C from equine heart, ubiquitin from bovine erythrocytes and myoglobin from equine skeletal muscle (all purchased from Sigma-Aldrich, St. Louis, Missouri, USA) with a final concentration of 10 µM in 50/49/1 H_2_O/acetonitrile/formic acid^83^. Acetonitrile (≥ 99.8%) and formic acid (99%) were purchased from Thermo Fisher Scientific (Waltham, Massachusetts, USA). Mass spectra were analysed and visualised using MassLynx V4.1 (Waters Corporation, Wilmslow, UK) and Origin 8 (OriginLab Corporation, Northampton, USA). Drift time profiles were generated by selecting the upper half of mass spectral peaks, i.e. the m/z range from half height to half height of each peak, to avoid overlap with adduct peaks.

## Supplementary information

Supplementary Information.

## Data Availability

Raw and processed data are available from the authors upon request.
