# Supplementary material for: Metal ions shape α-synuclein
Source: Sci Rep. 2020 Oct 1;10:16293. doi: 10.1038/s41598-020-73207-9 (PMC7529799; doi:10.1038/s41598-020-73207-9)
Supplement: Supplementary file 1 — Supplementary Information. [file 41598_2020_73207_MOESM1_ESM.pdf]

Supplementary information - Metal ions shape α-synuclein

Rani Moons[a,b], Albert Konijnenberg[a], Carl Mensch[c,d] Roos Van Elzen[e], Christian Johannessen[c], Stuart Maudsley[b], Anne-Marie Lambeir[e], *Frank Sobott[a,f,g]

a Biomolecular and Analytical Mass Spectrometry group, University of Antwerp, Antwerp, Belgium

b Receptor Biology Laboratory, University of Antwerp, Antwerp, Belgium

c Molecular spectroscopy group, University of Antwerp, Antwerp, Belgium

d Flemish Supercomputer Centre, Antwerp, Belgium

e Laboratory of Medical Biochemistry, University of Antwerp, Antwerp, Belgium

f Astbury Centre for Structural Molecular Biology, University of Leeds, Leeds, United Kingdom

g School of Molecular and Cellular Biology, University of Leeds, Leeds, United Kingdom

α-synuclein sequence

The 140 amino acid long sequence of α-syn can be divided into three major regions. The N-terminus contains a lot of positively charged lysine residues and this region is known to interact with negatively charged lipid headgroups present in biological membranes.[1-4] Upon membrane interaction, an amphipathic α-helix is formed. Familial mutations linked to Parkinson's disease also occur in this region.[5] In this region, metal ion binding sites are described for the sections of aa1-5 and aa48-52.[6] The middle region is the NAC region, containing mostly hydrophobic residues. This region can interact with NAC regions from other α-syn monomers because of hydrophobic interactions. This results in the formation of β-sheet structures, which are the building block of mature α-syn fibrils, while the termini keep their random coil conformation.[7] In this region are, to our knowledge, no metal ion binding sites determined. The C-terminus contains a high number of negatively charged residues, facilitating the interaction and coordination of various metal ions especially in the region of aa119-124.[6] Some important PTMs such as phosphorylation and ubiquitination can also occur here, as well as truncations. Truncated α-syn forms are also found *in vivo* and hence may play an important role in neurodegeneration.[8] The amino acid sequence, with negatively and positively charged residues indicated, divided into these three characteristic regions together with their most important properties is shown in Supplementary Figure S1.

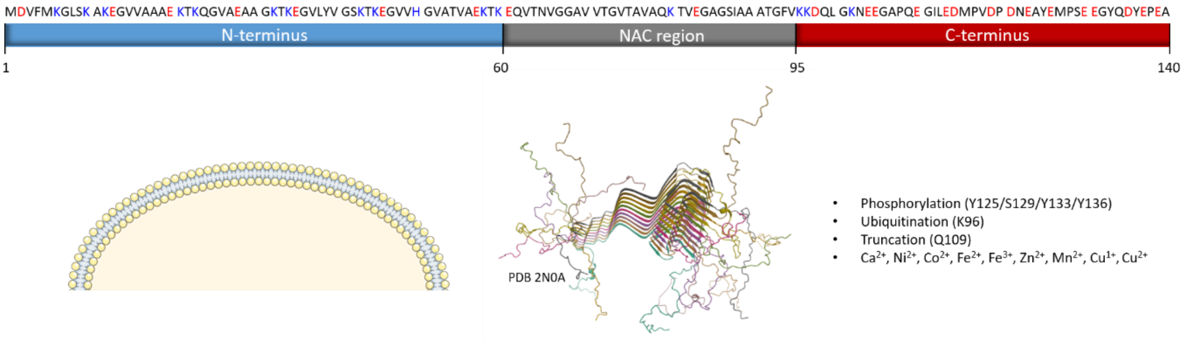

Figure S1

Drift time to collision cross section

Mass spectra recorded in ion mobility mode on the Synapt G2 HDMS contain information about the drift times of the ion present at every m/z peak. When selecting a specific peak (unbound or with a specific number of metal ions bound) in the mass spectrum (step 1 in Supplementary Figure S2), drift times of the ions present underneath that peak are retained (step 2 in Supplementary Figure S2). These drift times are still depending on the charge state of the analysed protein peak, so using denatured calibrants, with similar charge states and drift times as the protein of interest, drift time values are converted into CCS values, expressed in Å². These values can be compared across different charge states and mass spectra that were recorded using the same experimental settings.

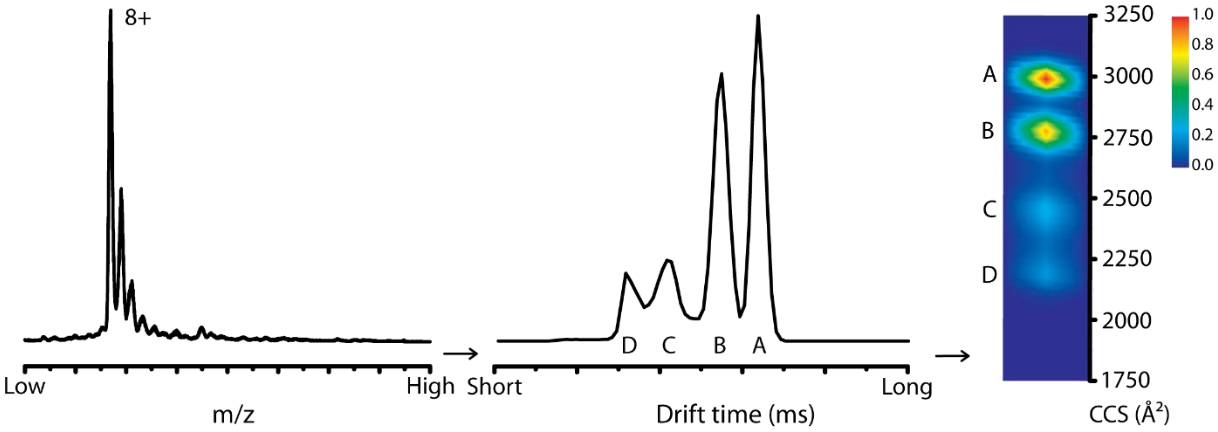

Figure S2

Bound vs. unbound α-synuclein

The percentage of unbound α-syn, and in this way indirectly the percentage of α-syn bound to metal ions, per metal salt and per ratio is plotted in a bar graph (Supplementary Figure S3) based on the 8+ peak of the corresponding mass spectrum. As expected, the percentage of unbound α-syn decreases per metal salt when higher salt concentrations are present, as long as the metal ion capacity of α-syn is not exceeded and the mass spectrum is of sufficient quality to determine binding stoichiometry.

With the addition of 1+ metal salts, there is always unbound α-syn detected, irrespective of the metal salt, even for a 1:250 protein to metal ion ratio. This might indicate that the binding of 1+ metal ions to α-syn is nonspecific and that the relative binding affinity is low for a higher number of 1+ metal ions. For 2+ metal ions, the unbound state disappears if the concentration of metal ions is high enough (except for Fe^{2+}). For Cu^{2+}, which is known to bind in a specific way to α-syn, there are already only bound states present when $CuCl_2$ is present in a 1:1 ratio. This indicates that with MS experiments a first classification can be made concerning the specificity and affinity of binding for certain ions and molecules. This can lead to important insights that can be translated to interactors and binding partners of α-syn *in vivo*. For $LaCl_3$ only 10% of the monomers are in a bound state when a 1:1 protein to metal ion ratio is used, while no unbound protein is detected for a 1:4 ratio. It is possible that this happens for other 3+ ions as well but that the ratios where this occurs give rise to too much interference in the mass spectrometer.

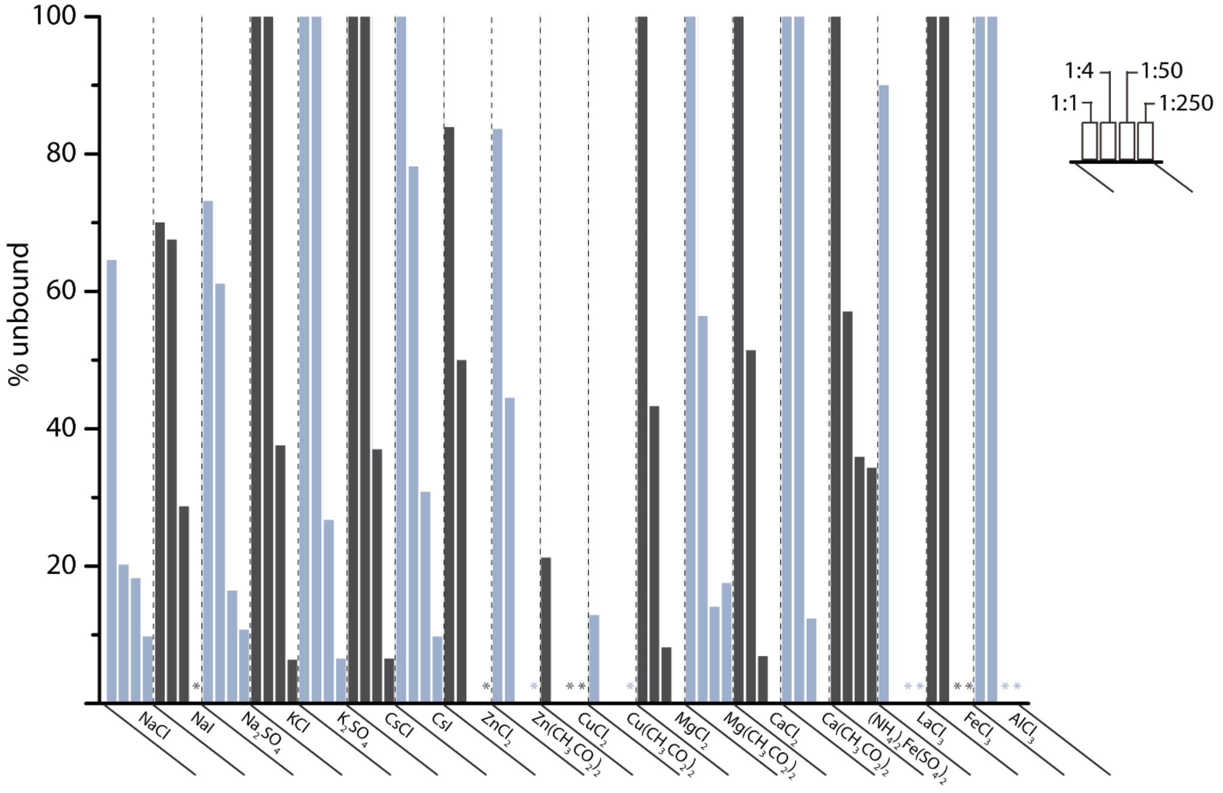

Figure S3

Mass spectra with metal ions present

When metal ions are present in the sample and binding to the protein, a possible shift of the charge state distribution in the mass spectra must be taken into account. However, it can be seen in Supplementary Figure S4 for various metal ions, which are representative per oxidation state for all other 1+, 2+ and 3+ metal ions that bind to the protein that the charge state range does hardly change

and always appears from around 5+ to 18+. For La^{3+} we can see a slight intensity shift towards lower charge states. No shift in charge state distribution indicates that metal ions replace protons and do not shift the whole charge state distribution to higher charge states when binding to the protein. Besides this, it also shows that there are still different conformations present at the same time, although IM-MS is needed to elucidate more subtle and detailed conformational changes. In Supplementary Figure S4 we enlarge the region of charge states 7+ to 9+, where metal ion binding stoichiometries are indicated. It is clear that for different charge states, the maximum number of binding and distribution of intensities of the bound peaks is almost identical, only varying in intensity of the overall charge state itself as could already be seen in Supplementary Figure S4.

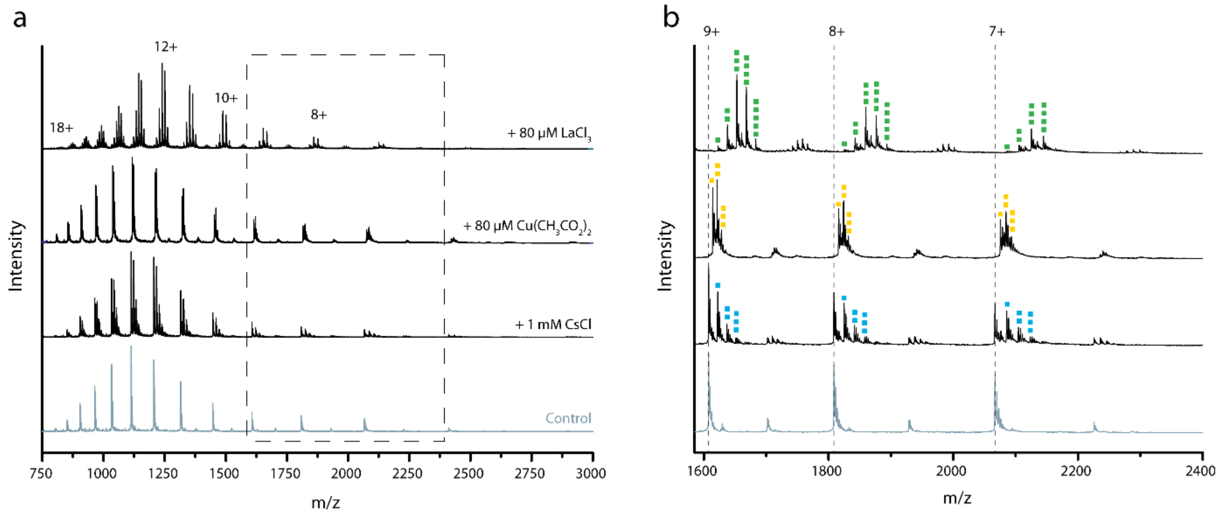

Figure S4

Intermediate charge states and globular proteins

The 8+ charge state was taken as a model for all intermediate charge states which behave in the same way upon metal ion binding. Supplementary Figure S5 displays the 7+, 8+ and 9+ charge state of α-syn in the presence of $ZnCl_2$ and shows that compaction of α-syn, when binding multiply charged metal ions, indeed does not only occur for the 8+ charge state. We here chose $ZnCl_2$ as an example and representative doubly charged metal ion to prove compaction occurs for different charge states. This is not only the case for $ZnCl_2$ but for all multiply charged metal ions (data not shown). These CCS plots can be compared with the CCS plots of the 7+, 8+ and 9+ charge state of lysozyme (Supplementary Figure S5). Lysozyme is a globular protein with a 3D structure stabilised by disulphide bridges and has a theoretical average molecular mass of 14307 Da, comparable to α-syn which has a theoretical average molecular mass of 14460.1 Da. For lysozyme there are no conformational changes detected upon binding of Zn^{2+} ions. As was the case for α-syn, no shifts in the charge state distribution are observed for lysozyme when metal ions are present or bound, again indicating that metal ions take the plce of protons to contribute to the total charge state. The detected conformational effects that were

seen for α-syn are thus specifically related to the IDP and not to a general effect that proteins would compact upon metal ion binding. This, on its turn, is again an indication that IDPs are more sensitive to conformational changes induces by charged interactors. Binding stoichiometries up to two Zn^{2+} ions are shown here, since this is the maximum number of ions bound to lysozyme.

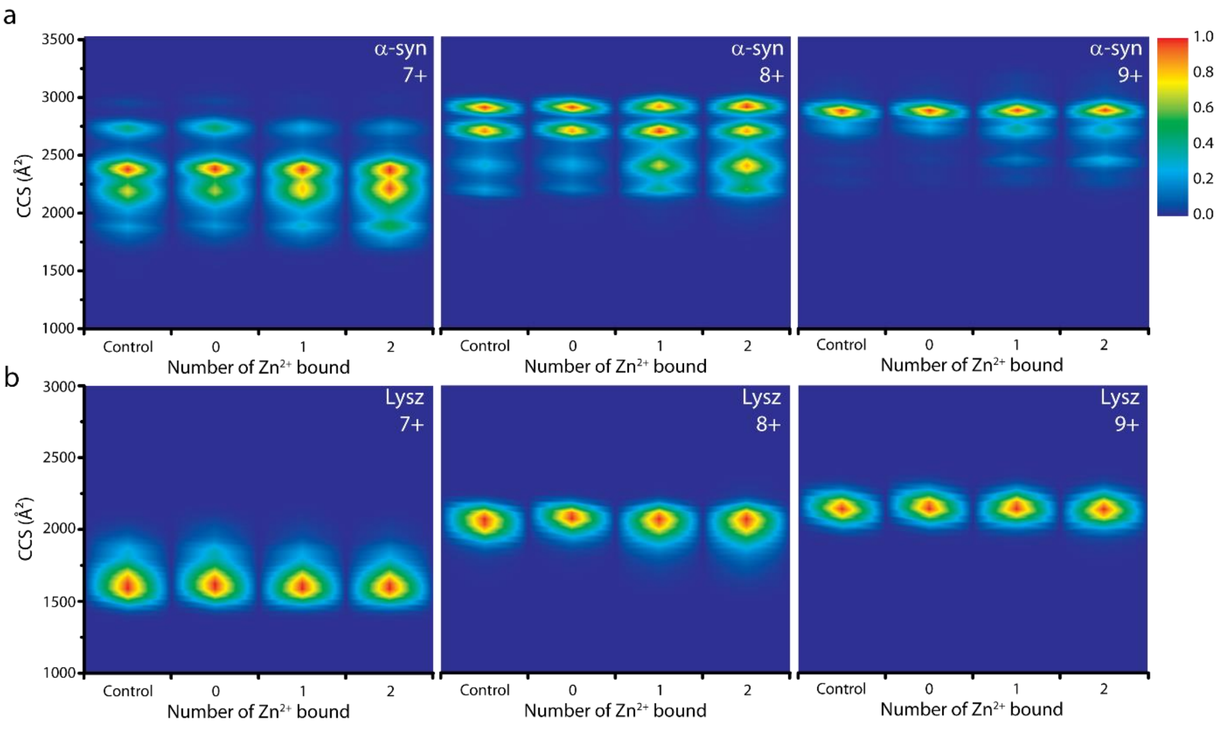

Figure S5

Singly charged metal ions

Unlike the compacting effect which occurs when multiply charged metal ions bind to α-syn, there is an intensity shift towards the most extended conformation (conformation "A") when 1+ metal ions are binding to the 8+ charge state of α-syn (Supplementary Figure S6). When NaCl is present but not bound to α-syn, the intensity of peak "A" and peak "B" are comparable. But when there are one or more Na^+ ions bound, the intensity of peak "A" increases. For K^+ and Cs^+ binding, similar effects were observed (data not shown).

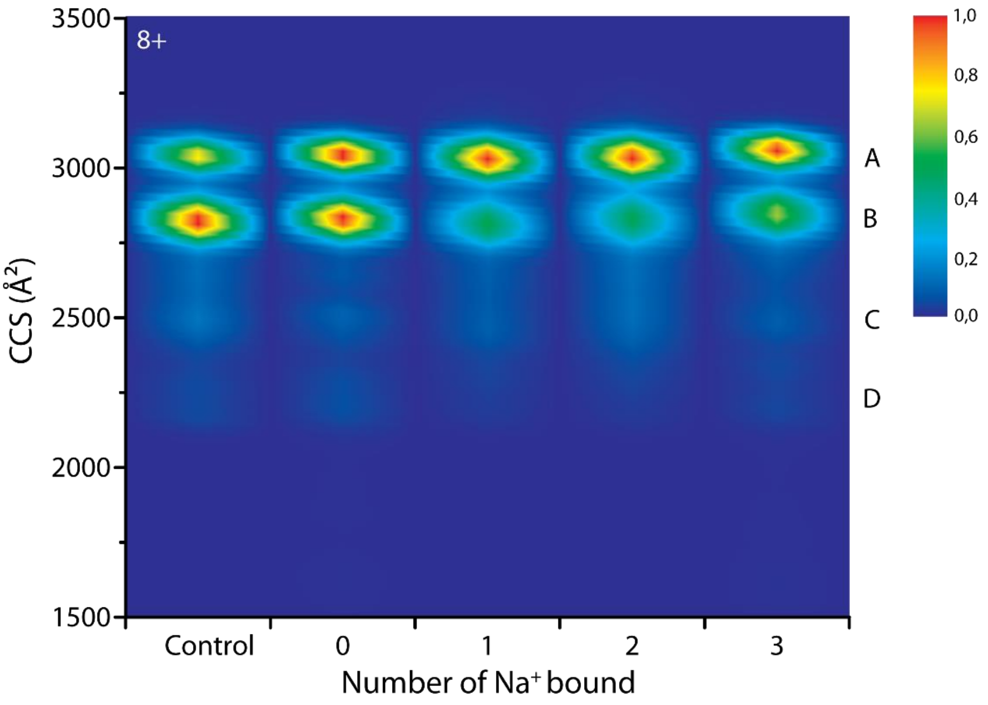

Figure S6

Circular dichroism spectroscopy

To investigate if the observed compaction upon multivalent ion binding to α-syn is potentially accompanied by formation of secondary protein structure, circular dichroism experiments are performed of α-syn in the presence of a 2+ and 3+ metal ion.

Lyophilised α-syn was dissolved in deionised H_2O as a 70 µM stock solution. $Cu(CH_3CO_2)_2$ and $LaCl_3$ were dissolved in deionised H_2O as 2 mM stock solution. In the final sample α-syn was diluted to a concentration of 7 µM in the presence of 80 µM metal salt with a final sample volume of 150 µL.

CD spectra were obtained using a Chirascan-plus CD spectrometer (Applied Photophysics, Leatherhead, UK) between 180 and 260 nm. A bandwidth of 1 nm was used, scan speed was set to 3 s/nm and the path length was 0.05 cm.

The resulting CD spectrum for $Cu(CH_3CO_2)_2$ and $LaCl_3$ (Supplementary Figure S7) shows a first maximum around 185 nm, followed by a minimum around 195-200 nm and a constant maximum starting around 215nm. This profile is similar to that of the α-syn control, indicating that the structure in solution is still predominantly disordered when metal ions are bound. This indicates compaction does not occur via formation of secondary protein structures.

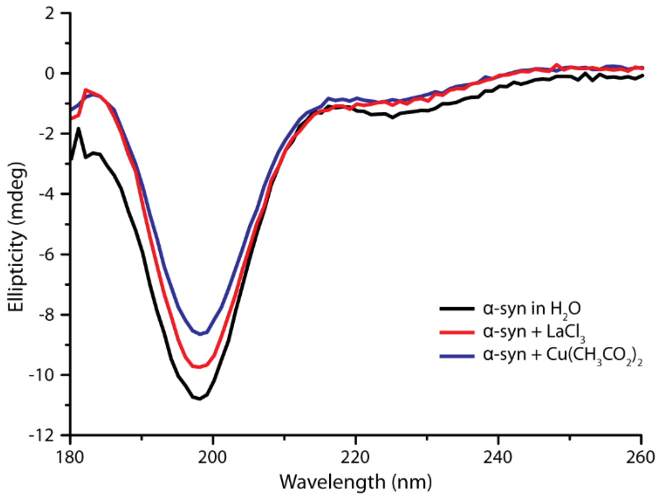

Figure S7

CCS comparison of different metal ions (8+ charge state)

Drift time plots of figure 6 (main text) can be converted into a CCS heat map after calibration (Supplementary Figure S8), which allows comparison of how the binding of, in this case, four metal ions alters the conformational space of α-syn. CCS fingerprints are grouped per metal ion oxidation state and compared to the control, *i.e.* conformations present for the 8+ charge state of α-syn monomers without metal ions present. It can be seen that for 1+ ions the intensity shifts to the most extended state, as was described for Na^+ before. For multiply charged ions compaction occurs, but with different conformational fingerprints for each type of metal ion.

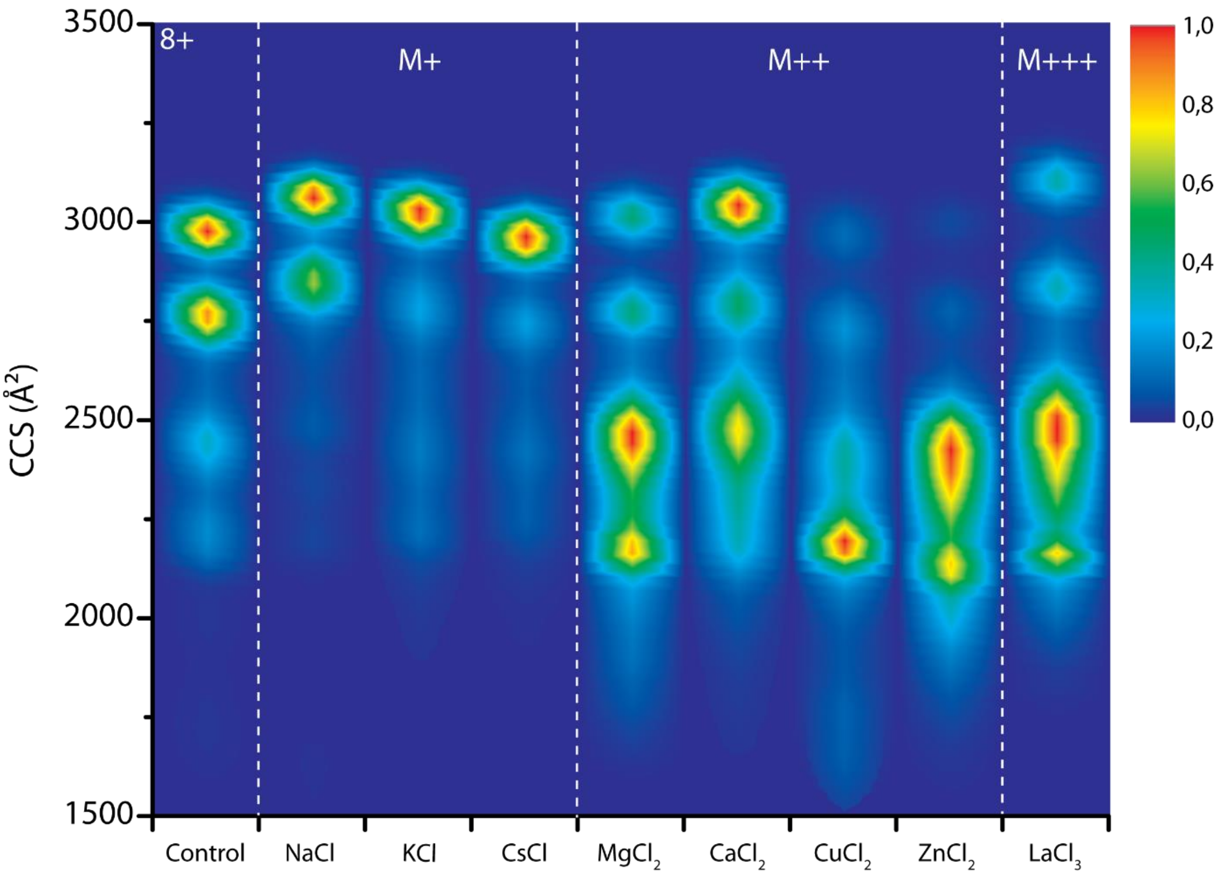

Figure S8

Effect of metal ion concentration on conformational change

In Supplementary Figure S9, drift time profiles are shown for the 8+ charge state of α-syn and when four Ca^{2+} ions are binding. The selected four Ca^{2+} bound state can be observed with different protein:metal ion ratios used. These drift time plots are extracted from the peaks in Figure 3 (main text) where four Ca^{2+} ions are bound to α-syn, i.e. from the 1:50; 1:100 and 1:250 protein:metal ion ratio spectra. The drift time profiles show a nearly identical distribution of conformational states highlighted as "A"-"D" with "A" the most extended and "D" the most compact state. The similar patterns indicate that irrespective of the excess of metal salt used, the binding sites and structural effects of metal coordination for that specific state are likely the same.

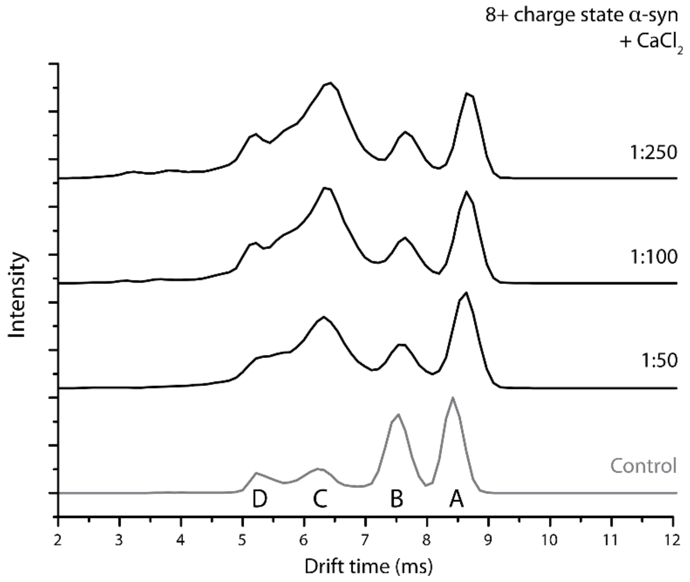

Figure S9

Effect of counterions

In addition to the effect of counterions when four metal ions bind to α-syn (Figure 9, main text), the same type of CCS plots in Supplementary Figure S10 include data with zero, one, two and three metal ions bound (top right, top left, bottom right and bottom left, respectively). For the unbound states with metal ions present in the sample ("0"), only very subtle counterion effects can be seen in the CCS plots. This indicates that when no metal ions are bound, there is no "memory" effect of counterions which were present in the sample but are lost during ESI. For metal-ions bound states, conformational differences due to the presence of a specific counterion become more and more clear. For both types of counterions, the trend towards more compaction with increased metal binding remains, but at different rates. For example, $Zn(CH_3COO)_2$ shows a pattern with one metal ion which matches $ZnCl_2$ with two metal ions bound. Only with the third metal ion does the chloride pattern catch up with the acetate. Related, but opposite observations can be made for Cu, where with one metal ion bound the most compact state is already found populated while the acetate only reaches a somewhat similar result with three metal ions. Again, the differences are more explicit for Cu^{2+} and Zn^{2+} binding, compared to Mg^{2+} and Ca^{2+} binding.

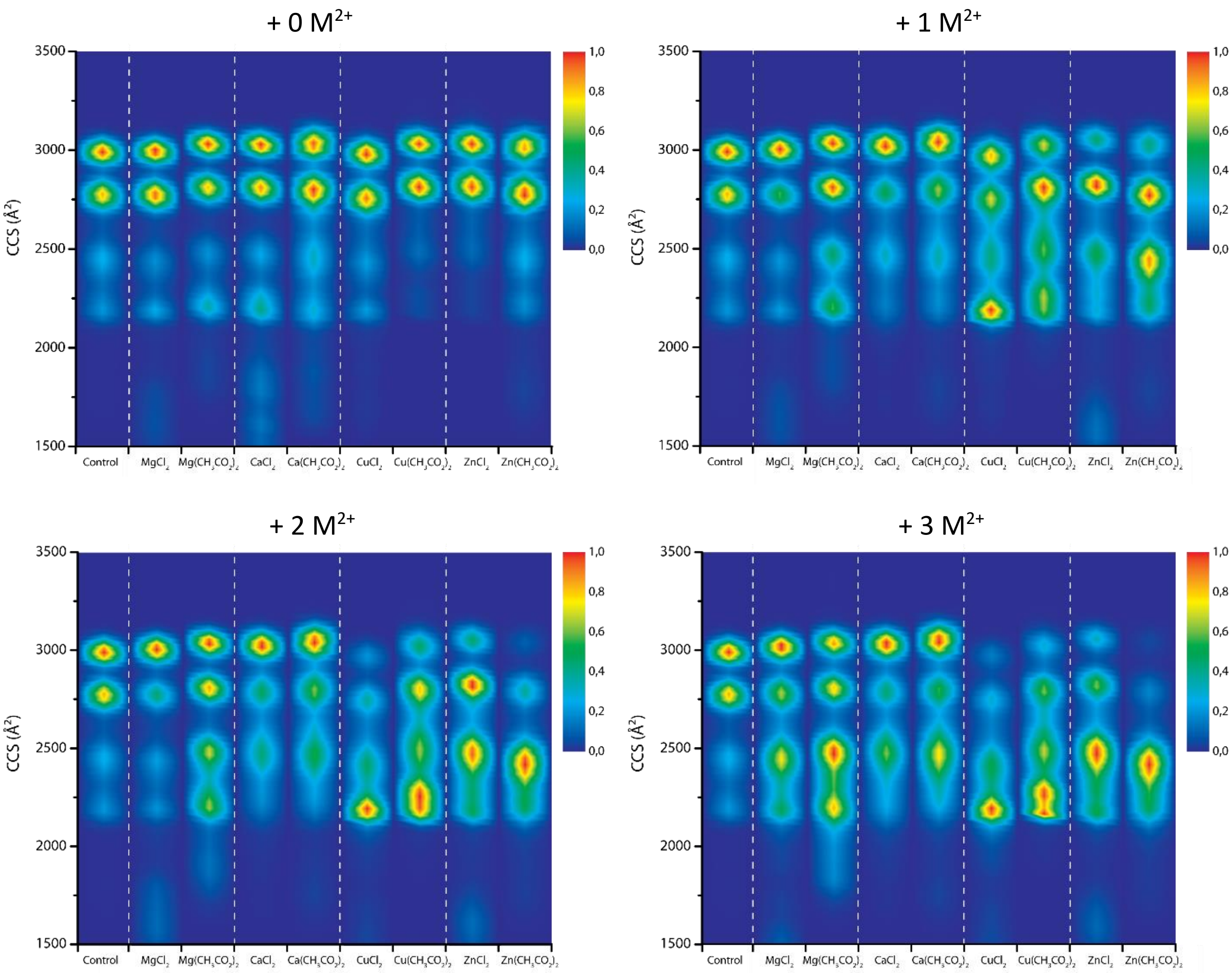

+ 0 M2+
+ 1 M2+
+ 2 M2+
+ 3 M2+
CCS (Å2)
3500
3000
2500
2000
1500
1,0
0,8
0,6
0,4
0,2
0,0
Control
MgCl2
Mg(CH3CO2)2
CaCl2
Ca(CH3CO2)2
CuCl2
Cu(CH3CO2)2
ZnCl2
Zn(CH3CO2)2

Figure S10

References

1 Binolfi, A. *et al.* Interaction of alpha-synuclein with divalent metal ions reveals key differences: a link between structure, binding specificity and fibrillation enhancement. *J Am Chem Soc* **128**, 9893-9901, doi:10.1021/ja0618649 (2006).

2 Lee, S. J. *et al.* Probing conformational change of intrinsically disordered alpha-synuclein to helical structures by distinctive regional interactions with lipid membranes. *Anal Chem* **86**, 1909-1916, doi:10.1021/ac404132g (2014).

3 Ueda, K. *et al.* Molecular cloning of cDNA encoding an unrecognized component of amyloid in Alzheimer disease. *Proc Natl Acad Sci U S A* **90**, 11282-11286, doi:10.1073/pnas.90.23.11282 (1993).

4 Shvadchak, V. V., Yushchenko, D. A., Pievo, R. & Jovin, T. M. The mode of alpha-synuclein binding to membranes depends on lipid composition and lipid to protein ratio. *FEBS Lett* **585**, 3513-3519, doi:10.1016/j.febslet.2011.10.006 (2011).

5 Flagmeier, P. *et al.* Mutations associated with familial Parkinson's disease alter the initiation and amplification steps of alpha-synuclein aggregation. *Proc Natl Acad Sci U S A* **113**, 10328-10333, doi:10.1073/pnas.1604645113 (2016).

6 Gonzalez, N. *et al.* Effects of alpha-synuclein post-translational modifications on metal binding. *J Neurochem* **150**, 507-521, doi:10.1111/jnc.14721 (2019).

7 Guerrero-Ferreira, R. *et al.* Cryo-EM structure of alpha-synuclein fibrils. *eLife* **7**, doi:10.7554/eLife.36402 (2018).

8 Prasad, K., Beach, T. G., Hedreen, J. & Richfield, E. K. Critical role of truncated alpha-synuclein and aggregates in Parkinson's disease and incidental Lewy body disease. *Brain Pathol* **22**, 811-825, doi:10.1111/j.1750-3639.2012.00597.x (2012).

9 Oueslati, A. Implication of Alpha-Synuclein Phosphorylation at S129 in Synucleinopathies: What Have We Learned in the Last Decade? *J Parkinsons Dis* **6**, 39-51, doi:10.3233/JPD-160779 (2016).

Figure legends

Supplementary Figure S1: Amino acid sequence of human wild-type α-syn. Amino acids with negatively charged side chains are in red, amino acids with positively charged side chains are in blue. The three different regions which can be distinguished and related specific properties are indicated and shown.[9]

Supplementary Figure S2: General workflow where per peak in the mass spectrum one or multiple conformations that are present at the same time are retained due to the intensity detected per drift time value. Using denatured ubiquitin, myoglobin and cytochrome C as calibrants, drift times can be converted into collision cross section (CCS) values expressed in Å². CCS values are plotted after normalization per charge or bound state, going from 1: very intense (red) to 0: not present (dark blue).

Supplementary Figure S3: Comparison of the percentage unbound α-syn and in this way indirectly the percentage of protein bound to one or more metal ions per metal salt per concentration of the metal salt. These results are based on the 8+ charge state of the mass spectra. Per metal salt four bars are shown, corresponding to 1:1, 1:4, 1:50 and 1:250 ratio from left to right respectively. Bars that correlate with mass spectra where it was difficult to determine the binding of metal ions because of a high noise level caused by the presence of the metal salt, are indicated with an asterisk.

Supplementary Figure S4: a: Full mass spectra of α-syn without metal ions present (control) and with Cs^{+}, Cu^{2+} or La^{3+} present and bound. This figure shows that no drastic charge state distribution shifts occur upon metal ion binding. b: Zoom of the 7+-9+ charge states region where peaks which can be attributed to different metal ion binding stoichiometries are clearly visible.

Supplementary Figure S5: CCS plots of the 7+, 8+ and 9+ charge state of a: α-syn and b: lysozyme, in the presence of $ZnCl_2$ (“0”) and bound to Zn^{2+}. The control lane shows the conformations present for the 8+ charge state of α-syn or lysozyme without metal ions present. For α-syn there is an intensity shift towards more compact conformations when Zn^{2+} binds, this occurs for every intermediate charge state. For lysozyme no conformational changes are detected when Zn^{2+} binds to the different charge states.

Supplementary Figure S6: CCS plot of conformations present for the 8+ charge state of α-syn when NaCl is present and when Na^{+} ions bind to the protein. The control shows the CCS plot for the 8+ charge state of α-syn without metal ions added. If NaCl is present there is already a slight intensity shift towards the most extended conformation, peak “A”, which is more drastic when Na^{+} is bound to α-syn. CCS values are plotted after normalization per charge or bound state, going from 1: very intense (red) to 0: not present (dark blue).

Supplementary Figure S7: Circular dichroism spectroscopy confirms that α-syn bound to multiply charged metal ions retains a largely random coil structure in solution, similar to the unbound control.

Supplementary Figure S8: CCS heat map of the 8+ charge state of α-syn monomers with four metal ions bound, compared to the conformational space of the control without metal ions present. An intensity shift towards the most extended state can be seen for 1+ metal ions, while multiply charged metal ions induce compaction of the IDP.

Supplementary Figure S9: Drift time profiles of the 8+ charge state of α-syn with four Ca^{2+}-ions bound with various $CaCl_2$ concentrations present in the sample (1:50; 1:100 and 1:250 protein:metal ion ratio). The drift time profiles are identical, indicating there is no conformational effect related to the final metal ion concentration in the sample. Different conformations are labelled “A” (most extended one) to “D” (most compact one).

Supplementary Figure S10: CCS plots showing the effect of specific counterions present when zero, one, two and three metal ions are bound (left to right, top to bottom respectively). Although the general pattern is consistent, small changes in CCS value are possible.
